# Stability Study of a Frozen Oseltamivir 15 mg/mL Oral Solution in Amber Glass Bottles

**DOI:** 10.3390/pharmaceutics18091130

**Published:** 2026-09-08

**Authors:** Juan Carlos Ruiz Ramirez, Adrián Gómiz Sáez, María Encarnación Martínez Madrid, Alice Charlotte Viney, José María Alonso Herreros, Pilar Almela Rojo

**Affiliations:** 1 Pharmacy Service, Los Arcos del Mar Menor University General Hospital, Murcian Health Service, 30739 San Javier, Murcia, Spain; alice.charlotte@carm.es (A.C.V.); josem.alonso@carm.es (J.M.A.H.); 2 Department of Pharmacology, Faculty of Pharmacy, CEIR Campus Mare Nostrum (CMN), University of Murcia, 30120 Murcia, Spain; palmela@um.es; 3 Biomedical Research Institute of Murcia Pascual Parrilla—IMIB, 30120 Murcia, Spain; 4 Pharmacy Service, Virgen de la Salud—Elda General Hospital, Valencian Health Service, 03600 Elda, Alicante, Spain; gomiz_adrian@gva.es; 5 Pharmacy Service, Santa Lucía University General Hospital, Murcian Health Service, 30202 Cartagena, Murcia, Spain; mariae.martinez48@carm.es

**Keywords:** oseltamivir, oral solution, stability, freezing, amber glass, HPLC

## Abstract

**Background/Objectives:** Oseltamivir is a widely used antiviral agent indicated for the treatment of influenza and plays a central role in pandemic preparedness strategies. Although an extemporaneously prepared 15 mg/mL oral solution is recognized in formularies, preparing large volumes during a pandemic requires extended storage options. However, no stability studies are currently available for an oseltamivir 15 mg/mL oral solution stored under freezing and subsequent refrigerated conditions for the duration of a standard treatment. The aim of this study was to evaluate the physicochemical and microbiological stability of a 15 mg/mL oseltamivir oral solution prepared from the active pharmaceutical ingredient (API) and packaged in amber glass containers. **Methods:** The oral solution was formulated using oseltamivir phosphate API, sodium benzoate as a preservative, and purified water and then packaged in 125 mL Type II amber glass bottles, allowing for thermal expansion. The samples were stored at −20 ± 2 °C for up to 75 days, followed by refrigerated storage (5 ± 3 °C) after thawing for up to 10 days. Chemical stability was assessed using a validated HPLC method in accordance with ICH guidelines and was defined as 90–110% recovery of the initial concentration. Physical stability (color, pH, particulate matter, crystallization, and homogeneity) and microbiological stability were also evaluated. **Results:** The HPLC method demonstrated excellent linearity, precision, and accuracy. Oseltamivir concentrations remained within the predefined acceptance limits throughout the 75-day study period under freezing conditions, with no significant changes in pH, color, or particulate formation. After thawing, the drug concentration continued to remain fully stable and within the required limits for up to 10 days under refrigerated conditions. Despite the prolonged storage and phase changes, no significant changes in physical parameters were observed, and microbiological testing confirmed the absence of aerobic, anaerobic, and fungal microorganisms on the final day of the study. **Conclusions:** Oseltamivir 15 mg/mL oral solution in amber glass bottles is physicochemically and microbiologically stable for up to 85 days (75 days under frozen conditions plus 10 days under refrigeration after thawing).

## 1. Introduction

**Figure 1:**
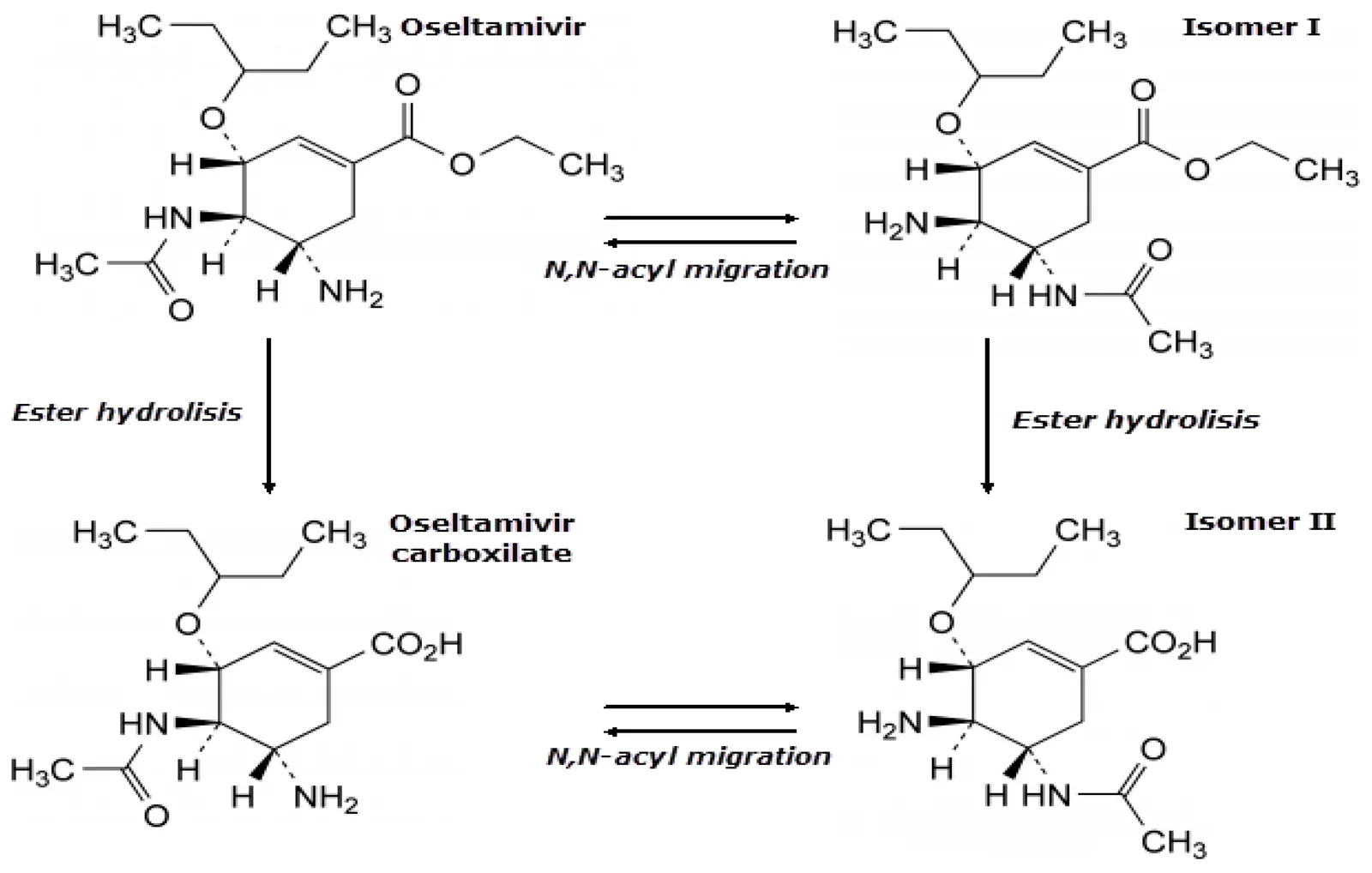
Structure of oseltamivir phosphate and chemical transformation pathways.

Oseltamivir phosphate, ethyl (3R,4R,5S)-4-acetylamino-5-amino-3-(1-ethylpropoxy)-1-cyclohexene-1-carboxylate phosphate (Figure 1), is a white or almost white powder, freely soluble in water and methanol, and practically insoluble in methylene chloride [1,2]. It is an orally active prodrug widely indicated in adults and children, including term newborns, for the treatment and prophylaxis of influenza A and B infections [3,4,5].

Upon oral administration, the molecule is rapidly absorbed and undergoes extensive hepatic biotransformation via carboxylesterases to yield its active metabolite, oseltamivir carboxylate [4,5,6]. This metabolite selectively inhibits the viral neuraminidase glycoprotein on the surface of the virion, preventing the cleavage of sialic acid residues and subsequently blocking the release and dissemination of newly formed viral particles [4,5,7].

During pandemic outbreaks—such as the 2009 A(H1N1) influenza crisis—and seasonal surges, the demand for oral liquid pediatric formulations of oseltamivir becomes critical, as capsules are unsuitable for children, elderly patients, or individuals with swallowing difficulties [8]. In such scenarios, public and hospital pharmacies are designated to prepare extemporaneous oral preparations [9]. However, preparing these formulations on a patient-by-patient basis during a health emergency poses severe logistical and time-consuming bottlenecks, highlighting the urgent need for robust, long-term stockpiling strategies [10,11].

The German National Formulary (DAC/NRF) includes a monograph entitled “Oseltamivir Oral Solution 15 mg/mL for Adults or for Children (DAC/NRF 31.2)”, compounded from oseltamivir active pharmaceutical ingredient (API) as oseltamivir phosphate, with purified water and sodium benzoate as an antimicrobial preservative [12,13]. Its physical appearance should be that of a clear, colorless, homogeneous liquid, free of suspended particles or visible turbidity. This characteristic transparency visually distinguishes the DAC/NRF 31.2 solution from suspensions reconstituted from commercial capsules or marketed oral suspensions, which do exhibit an opaque, white-to-yellowish appearance due to the presence of insoluble excipients in an aqueous vehicle such as xanthan gum, titanium dioxide, and tutti-frutti flavoring [5,14].

The primary barrier to developing stable, pre-prepared liquid formulations of oseltamivir lies in its intrinsic chemical instability in aqueous environments [6,15]. In solution, oseltamivir phosphate undergoes a characteristic biexponential decay across the pH range of 2.0 to 7.0. This decomposition is governed by two simultaneous, distinct pathways: the irreversible hydrolysis of the ethyl ester group, which produces oseltamivir carboxylate, and a reversible, intramolecular N,N-acyl migration of the acetyl moiety from the C-4 acetamido group to the adjacent C-5 primary amine, yielding the toxicologically restricted degradant known as Isomer I (Figure 1). Both oseltamivir carboxylate and Isomer I can undergo subsequent degradation to form Isomer II through parallel acyl migration and ester hydrolysis, respectively (Figure 1) [6].

The rates of these degradation pathways are highly dependent on pH and temperature. Oseltamivir exhibits a V-shaped pH-rate profile with maximal chemical stability situated at pH 4.0 [6]. At pH values above and below this optimum, the rate of ester hydrolysis increases linearly with a slope of approximately unity, indicating specific acid- and base-catalyzed degradation [6]. In alkaline or neutral media, oseltamivir decomposes rapidly; for example, the use of potable water (typically pH ~7.4) instead of purified water accelerates hydrolytic degradation, reducing the solution’s shelf life to only 14 days and provoking the physical precipitation of insoluble calcium and magnesium phosphates [13]. This degradation can be suppressed by adding 0.1% anhydrous citric acid to buffer the formulation to an optimal pH of 4.2 [13]. Furthermore, the primary amine of oseltamivir makes the molecule susceptible to Maillard browning reactions when formulated alongside reducing sugars (such as glucose, fructose, or galactose), causing unacceptable darkening and accelerated chemical loss under elevated thermal stress [8].

Another important issue is the type of container used to hold the medicine ready for administration. In pharmaceutical terms, glass containers intended to hold medicinal solutions and suspensions are usually characterized by the following: high chemical inertness, which minimizes interactions between the medicine and the container; high hydrolytic resistance, especially in Type I glass, making them suitable for solutions, suspensions, and parenteral preparations; low permeability to gases and water vapor, which helps protect the formulation against oxidation and hydrolysis; transparency or amber/topaz coloration, depending on the need for visual inspection or light protection, particularly in light-sensitive formulations; the ability to undergo sterilization and, when required, depyrogenation, which is essential for containers intended for sterile solutions; good mechanical and dimensional resistance, allowing for safe filling, sealing, and transport; and compatibility with different closure systems, such as elastomeric stoppers and aluminum caps for vials or polypropylene caps for bottles, which help maintain container closure integrity [16,17].

The stability of oseltamivir oral solutions and suspensions has been evaluated under refrigerated and room-temperature conditions, containing different concentrations of oseltamivir [8,9,13,15,18]. However, there are no stability studies of a frozen aqueous solution of oseltamivir 15 mg/mL, and the available evidence does not adequately address the effect of freezing and subsequent thawing on an aqueous solution prepared from oseltamivir phosphate API. In particular, the potential consequences of ice crystallization, solute concentration during freezing, redispersibility after thawing, and refrigerated storage during the treatment period remain insufficiently characterized.

Therefore, the aim of this study was to evaluate the physicochemical and microbiological stability of a prepared oseltamivir 15 mg/mL oral solution, packaged in Type II amber glass bottles, under a dual-phase preservation protocol: an extended frozen storage phase (−20 °C) for up to 75 days, followed by by a post-thaw refrigerated storage phase (5 °C) for up to 10 days. 

## 2. Materials and Methods

### 2.1. Preparation of Oseltamivir 15 mg/mL Oral Solution

The compounding process was performed using oseltamivir phosphate API (Roche Farma, Madrid, Spain) and sodium benzoate API (Acofarma Distribución, S.A., Madrid, Spain), which were reconstituted with purified water obtained from a Milli-Q^®^ Integral 3/5/10/15 system (Merck KGaA, Darmstadt, Germany). Additionally, 125 mL Type II amber glass bottles, designed to allow for expansion upon freezing, were used to store the preparation (José Mestre, S.A., Barcelona, Spain).

Three batches of the oral solution bottles containing 50 mL of the formulation were prepared according to the DAC/NRF (Table 1) and subsequently frozen (frozen stability group (FSG). The volume of oral solution prepared for each batch was 500 mL. The required amount of oseltamivir phosphate (9.855 g) was weighed to obtain a solution containing 7.5 g of oseltamivir in a final volume of 500 mL. Subsequently, 0.5 g of sodium benzoate was weighed. Sodium benzoate was dissolved in 250 mL of purified water under magnetic stirring at 400 rpm. Oseltamivir phosphate was then gradually incorporated under the same stirring conditions until a homogeneous solution was obtained. Finally, 50 mL of the oral solution was transferred into the amber glass bottles [12].

On the day of analysis, bottles assigned to the FSG were removed from the freezer and allowed to thaw at room temperature (22 ± 2 °C) for 1 h; bottle integrity was subsequently assessed by visual inspection. Subsequently, they were subjected to a gentle rocking motion for two minutes. After sampling, they were transferred to the refrigerated stability group (RSG), where they were analyzed on the days specified in the analytical protocol; in this case, the bottles were left at room temperature (22 ± 2 °C) for 15 min, and then they were gently rocked for 2 min. The freezing conditions were maintained at −20 ± 2 °C in the FSG, and the refrigeration conditions were set at 5 ± 3 °C in the RSG.

The preparation process was carried out in accordance with the specific recommendations included in the Guide for Good Preparation Practices of Medicines in Hospital Pharmacy Services (GPPMHPS) [19]. Freezing was selected as a non-routine, exploratory storage condition to assess stability during advance preparation and thawed refrigerated holding rather than to simulate standard conservation conditions for patient use.

### 2.2. Chromatographic Method

A Waters Breeze HPLC system (Waters Cromatografía, S.A., Barcelona, Spain) equipped with an XBridge 5 µm C18 reversed-phase column (Waters Cromatografía, S.A., Barcelona, Spain) was used. Chromatographic separation was performed under isocratic conditions using a mobile phase composed of acetonitrile/methanol/6.8 g/L potassium dihydrogen phosphate in water for chromatography (13.5/24.5/62, *v*/*v*), with the pH adjusted to 6 using 1 M potassium hydroxide. The flow rate was set at 1.2 mL/min, detection was carried out at 207 nm, the column temperature was maintained at 50 °C, the injection volume was 15 µL, and the total run time was 34 min [1]. HPLC-grade acetonitrile, methanol, and phosphoric acid were purchased from Panreac Química S.L.U. (Barcelona, Spain), and ultrapure water was obtained using the Milli-Q^®^ Integral 3/5/10/15 system (Merck KGaA, Darmstadt, Germany). The oseltamivir phosphate (impurity B-free) reference standard product was obtained from Merck Life Science, S.L. (Madrid, Spain).

Method validation: The assay method described in the monograph for oseltamivir phosphate in the European Pharmacopoeia [2] was used as incorporated into the Spanish Royal Pharmacopoeia [1] and validated for linearity, precision, and accuracy in accordance with ICH Q2 (R1) guidelines [20]. The European Pharmacopoeia assay for oseltamivir phosphate is considered specific within the pharmacopeial framework to ensure selectivity [2].

Linearity between peak area and oseltamivir concentration was assessed across eight calibration levels from 0.3 to 1.0 mg/mL (0.3, 0.4, 0.5, 0.6, 0.7, 0.8, 0.9, and 1.0 mg/mL). A calibration curve was constructed by linear regression to determine the coefficient of determination (R^2^), slope (a), and y-intercept (b) [21]. Precision was evaluated through intra- and inter-day repeatability studies. The intra-day assessment involved five replicate analyses on the same day at 80%, 100%, and 120% of the target concentration (0.6 mg/mL), whereas the inter-day study comprised two replicates over four different days at the same relative concentrations. Mean values, standard deviations, and coefficients of variation were calculated, with acceptance criteria of less than 1% for intra-day and less than 2% for inter-day repeatability [21]. Accuracy was determined by recovery studies performed in triplicate at oseltamivir concentrations of 0.4, 0.6, and 0.8 mg/mL. Recovery percentages were calculated and compared with the theoretical value (100%) using Student’s *t*-test [21,22]. The limits of detection (LOD) and quantification (LOQ) for oseltamivir were calculated from the standard deviation of the response (σ) and the slope (S) of the calibration curve, using the equations LOD = 3.3 × σ/S and LOQ = 10 × σ/S, in accordance with ICH recommendations [20,21,22]. Nevertheless, this study did not include dedicated forced-degradation experiments to formally establish the absence of interference from degradation products. For this reason, on each day of analysis, an oseltamivir standard solution (15 mg/mL) was injected in triplicate. Quantification of oseltamivir in both the standard and the sample was performed using an external standard calibration curve. Peak identity was confirmed by retention time matching, and sample concentration was determined by interpolation on the calibration curve, allowing for estimation of the percentage of oseltamivir remaining in the sample.

### 2.3. Chemical Stability

**Figure 2:**
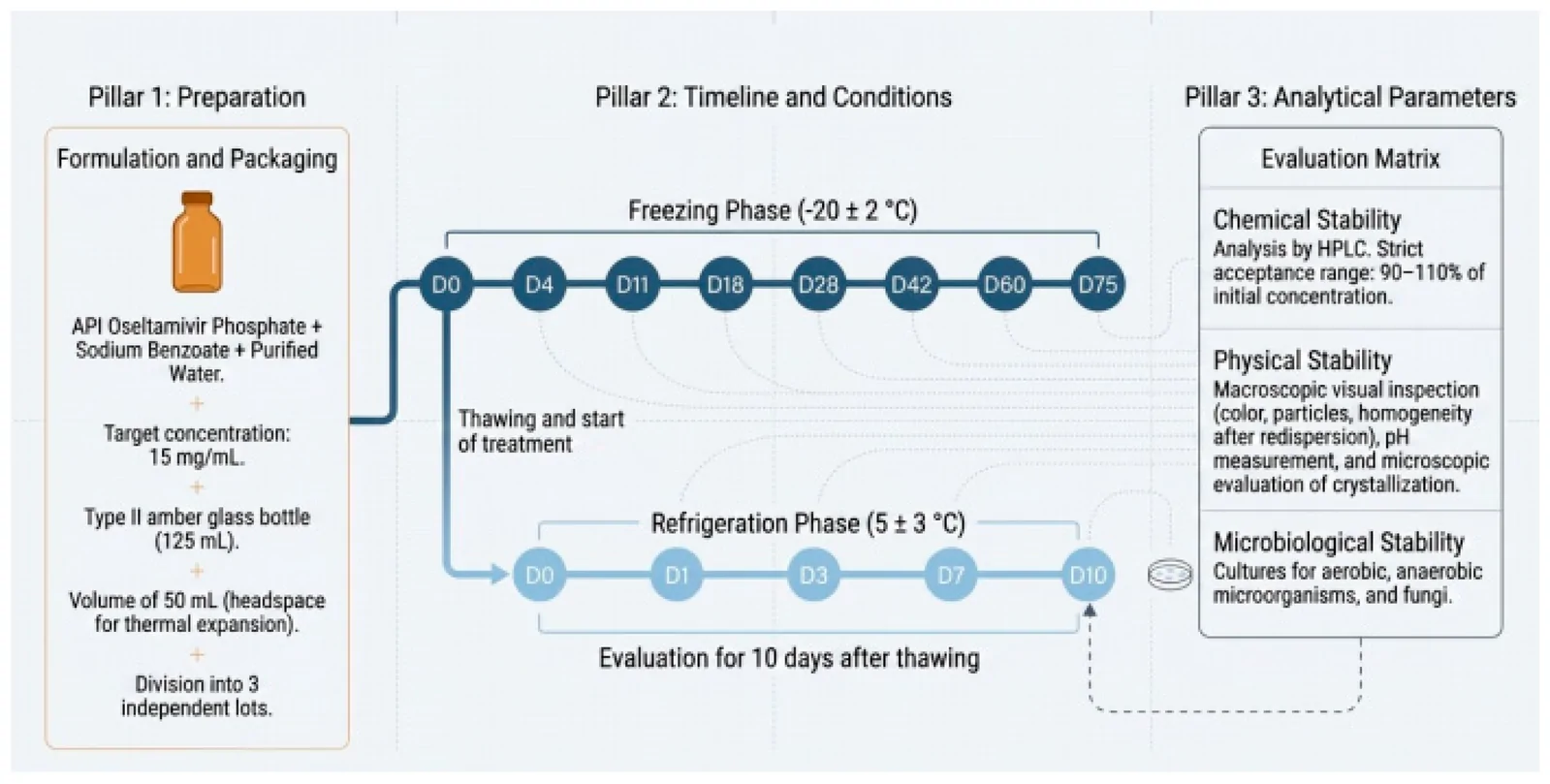
Study design scheme.

The chemical stability of the oseltamivir 15 mg/mL oral solution was evaluated over 75 days in the FSG (days 0, 4, 11, 18, 28, 42, 60, and 75) and over 10 days in the RSG after thawing (days 0, 1, 3, 7, and 10) (Figure 2).

On each analysis day, a 1 mL sample was taken from each batch of oseltamivir oral solution 15 mg/mL and diluted to 25 mL with the mobile phase (theoretical oseltamivir concentration: 0.6 mg/mL = target concentration).

The formulation was considered stable if the drug concentration remained within 90–110% of the initial concentration throughout the 75 days in the FSG and the 10 days in the RSG [21,23,24].

### 2.4. Physical Stability

Physical stability was evaluated for each batch by macroscopic visual inspection: color, visible particulates, and appearance of the solution after redispersion (in the case of thawing in the FSG). pH measurement and microscopic assessment of crystallization were also verified. On the predefined study days, 5 mL aliquots from each formulation were collected and inspected for particulate matter, crystal formation, turbidity, precipitation, and color changes. pH was determined using a calibrated SevenMulti™ pH meter (Mettler Toledo, Cornellà de Llobregat, Spain), while particle and crystal detection was performed using a visual inspection station with black-and-white backgrounds under bright-field illumination (CleanviewTM Inspection, Sterigene-Synexin, Francoville, France) in combination with a SediMAX2™ phase-contrast microscope (77 Elektronika, Budapest, Hungary).

### 2.5. Microbiological Stability

Microbiological stability was evaluated for each batch on the assessment day, corresponding to day 10 after thawing and refrigerated storage. By that time, the bottles had been opened on four occasions to obtain the samples scheduled in accordance with the study design. The samples were then inoculated into a culture medium for aerobic bacteria and fungi (BD BACTEC™ Peds Plus™/F, Becton Dickinson and Company, San Agustín de Guadalix, Spain) and into a specific medium for anaerobic bacteria (BD BACTEC™ Lytic/10 Anaerobic/F, Becton Dickinson and Company, San Agustín de Guadalix, Spain).

Culture media were incubated for 5 days at 35 ± 1.5 °C in a CO_2_-enriched atmosphere using the BD BACTEC™ fluorescent series system (Becton Dickinson and Company, San Agustín de Guadalix, Spain). The system detects microbial growth indirectly by monitoring CO_2_ production, which is associated with microbial metabolism. Each culture vial contains a chemical sensor that responds to increases in CO_2_ concentration. The instrument automatically monitors the sensor every 10 min and measures changes in fluorescence, which are proportional to the amount of CO_2_ generated. An increase in fluorescence is interpreted as a presumptive indication of viable microorganisms in the vial. Because positive and negative vials do not necessarily show a visible difference in the sensor itself, the final interpretation is based primarily on the instrument reading. After completion of the 5-day incubation period, a vial was considered negative when no significant increase in fluorescence, reflecting CO_2_ production, had been detected and no visible signs of microbial growth—such as turbidity, hemolysis, or bulging of the septum—were present.

Each box of culture media was supplied with its corresponding quality-control certificates, which specified the test microorganisms, including the ATCC^®^ strains listed in the CLSI M22 standard (Quality Control for Commercially Prepared Microbiological Culture Media): Streptococcus pyogenes ATCC^®^ 19615, Escherichia coli ATCC^®^ 25922, Streptococcus pneumoniae ATCC^®^ 6305, Pseudomonas aeruginosa ATCC^®^ 27853, Candida albicans ATCC^®^ 18804, Neisseria meningitidis ATCC^®^ 13090, Alcaligenes faecalis ATCC^®^ 8750, Haemophilus influenzae ATCC^®^ 19418, and Staphylococcus aureus ATCC^®^ 25923.

If the BD BACTEC™ system detected microbial growth, the European Pharmacopoeia tests would be performed to determine compliance with the acceptance criteria for the microbiological quality of non-sterile dosage forms, specifically aqueous preparations for oral use [25,26].

## 3. Results

### 3.1. Validation of the Analytical Method

The HPLC method demonstrated excellent linearity across the tested range, with a coefficient of determination ($R^{2}$) of 0.9992 and a regression equation of y=23,691,246.380x+62,139.288 (Supplementary Materials, Table S1 and Figure S1). Both intra-day and inter-day precision for the three oseltamivir quality control levels were highly satisfactory: intra-day relative standard deviations (RSD%) ranged from 0.264% to 0.596% (Table 2), while inter-day RSD% ranged from 0.378% to 0.608% (Table 3). These values successfully met ICH criteria for repeatability (≤1%) and intermediate precision (≤2%), respectively (Supplementary Materials, Tables S2 and S3). Method accuracy, determined by recovery rates at three concentration levels, ranged from 99.425% to 100.700%, falling consistently within the 98–102% acceptance interval (Supplementary Materials, Table S4). The LOD and LOQ were established at 0.030 mg/mL and 0.100 mg/mL, respectively.

**Table 2:** Intra-day repeatability of oseltamivir 15 mg/mL oral solution samples at 67, 100, and 133% of the target concentration of 0.6 mg/mL.

Theoretical Concentration (mg/mL)	0.4 (67%)	0.6 (100%)	0.8 (133%)
Mean (mg/mL)	0.399	0.599	0.800
SD	0.001	0.003	0.005
RSD%	0.264	0.522	0.596
Accuracy%	99.750	99.793	100.038

RSD%: relative standard deviation; SD = standard deviation.

**Table 3:** Inter-day repeatability of oseltamivir 15 mg/mL oral solution samples at 67, 100 and 133% of the target concentration of 0.6 mg/mL.

Theoretical Concentration (mg/mL)	0.4 (67%)	0.6 (100%)	0.8 (133%)
Mean (mg/mL)	0.399	0.600	0.802
SD	0.002	0.004	0.005
RSD%	0.378	0.712	0.608
Accuracy%	99.848	100.020	100.232

RSD%: relative standard deviation; SD = standard deviation.

**Figure 3:**
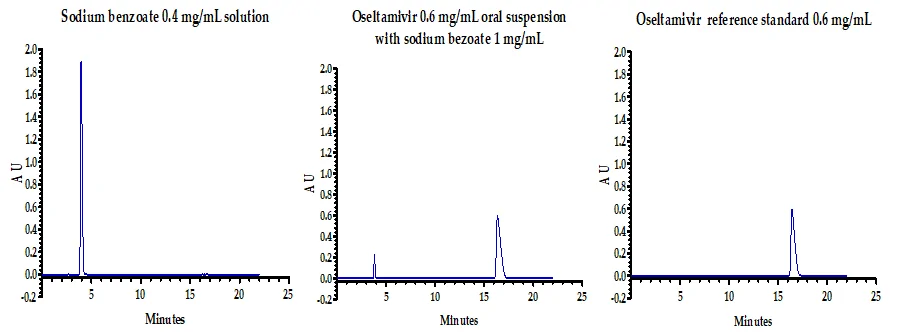
Chromatograms of sodium benzoate, oseltamivir oral solution, and oseltamivir reference standard to characterize the sodium benzoate peak.

The oseltamivir 15 mg/mL oral solution contains sodium benzoate as a preservative agent. This compound exhibits absorption at 207 nm, which accounts for the appearance of a corresponding peak (around minutes 4–5) in the chromatograms of oseltamivir 0.6 mg/mL oral solution. Therefore, a 0.4 mg/mL sodium benzoate solution was prepared in the mobile phase and compared with the chromatogram of the 0.6 mg/mL oseltamivir oral solution, which contains sodium benzoate at a concentration of 0.04 mg/mL, and with that of a 0.6 mg/mL oseltamivir solution prepared from the oseltamivir phosphate reference standard. The absorption peak observed around minutes 4–5 increased considerably, as can be seen in Figure 3, so it was confirmed that this peak corresponded to sodium benzoate.

### 3.2. Stability Study

#### 3.2.1. Freezing Conditions

**Figure 4:**
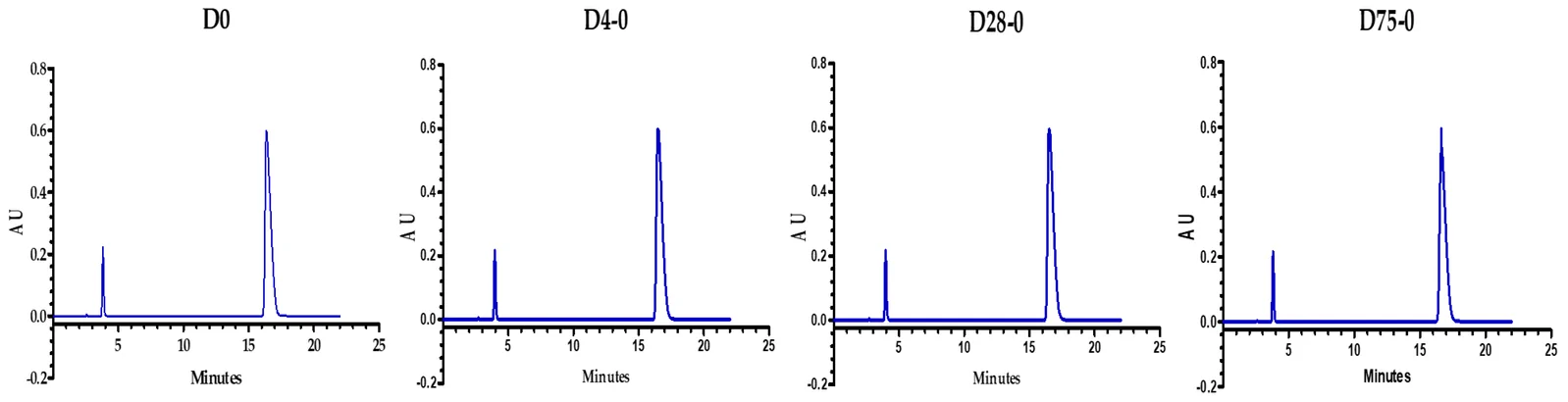
Chromatograms of oseltamivir 15 mg/mL oral solution in amber glass bottles under freezing conditions on days D0, D4-0, D28-0, and D75-0 (D + Number1 or D + Number1-Number2; Number 1 = day of thawing and transfer to refrigerated group; Number 2 = day of test in each refrigerated group).

**Figure 5:**
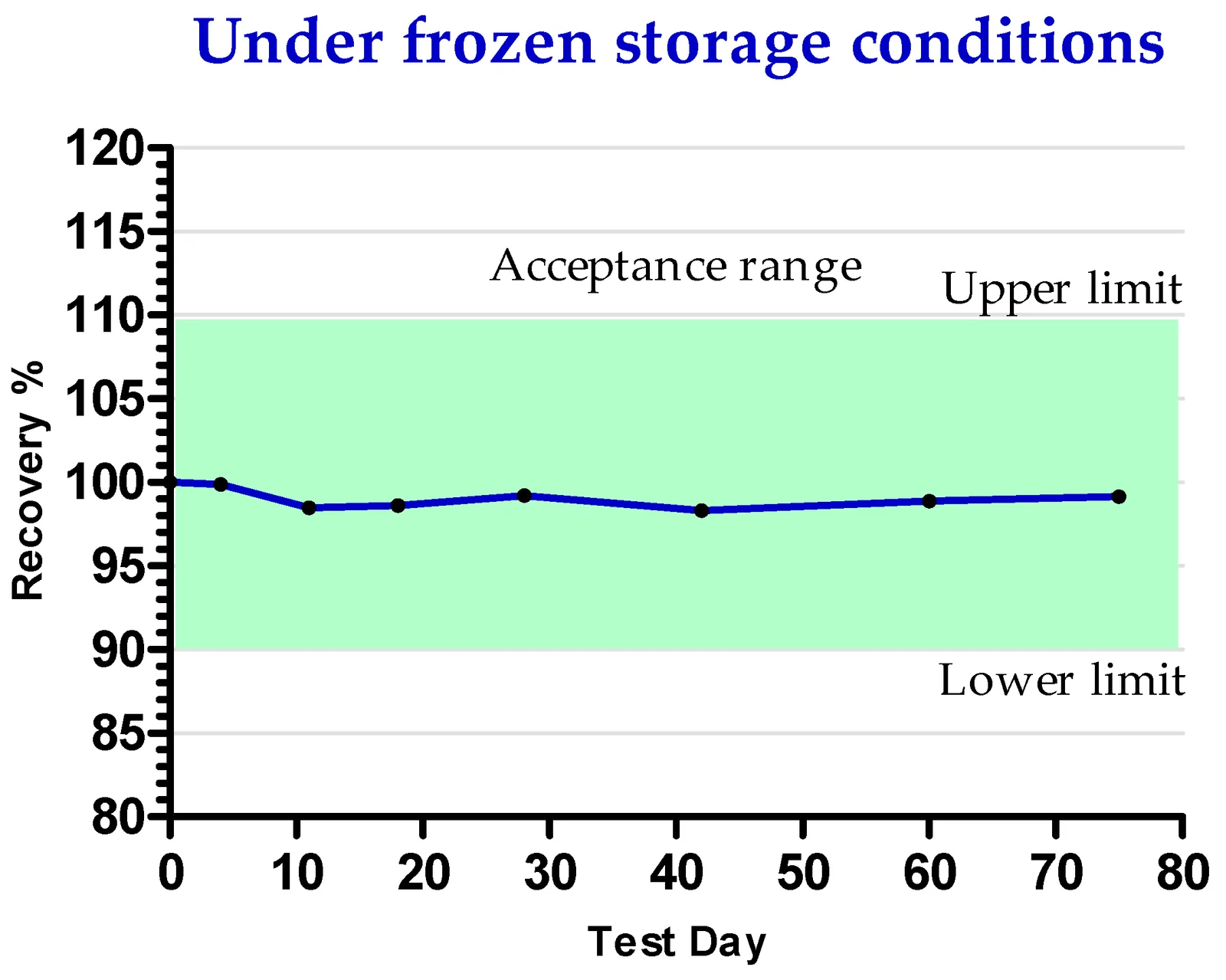
Evolution of percentage recovery of oseltamivir 15 mg/mL oral solution in amber glass bottles.

The chemical stability of the formulation was evaluated by quantifying oseltamivir concentrations in amber glass bottles at each scheduled sampling time point, using the procedure described previously. Mean concentrations were expressed as percentage recovery relative to the initial value, with D0 defined as 100%, as reported in Table 4. Representative chromatograms obtained at D0 and on the test days D4, D28, and D75 for the formulation stored under freezing conditions are presented in Figure 4. The results demonstrated that oseltamivir concentration remained within the predefined acceptance criteria throughout the 75-day storage period under those conditions (Table 4 and Figure 5). Full concentration data are provided in the Supplementary Materials (Table S5).

During the study period, no changes were observed in visual appearance, including color and turbidity, nor in the presence of visible particulates or crystallization, and pH values remained within the acceptable range (Table 4).

The 15 mg/mL oseltamivir oral solution stored in amber glass bottles under freezing conditions was consequently considered physically and chemically stable for up to 75 days.

#### 3.2.2. Refrigerated Conditions

**Figure 6:**
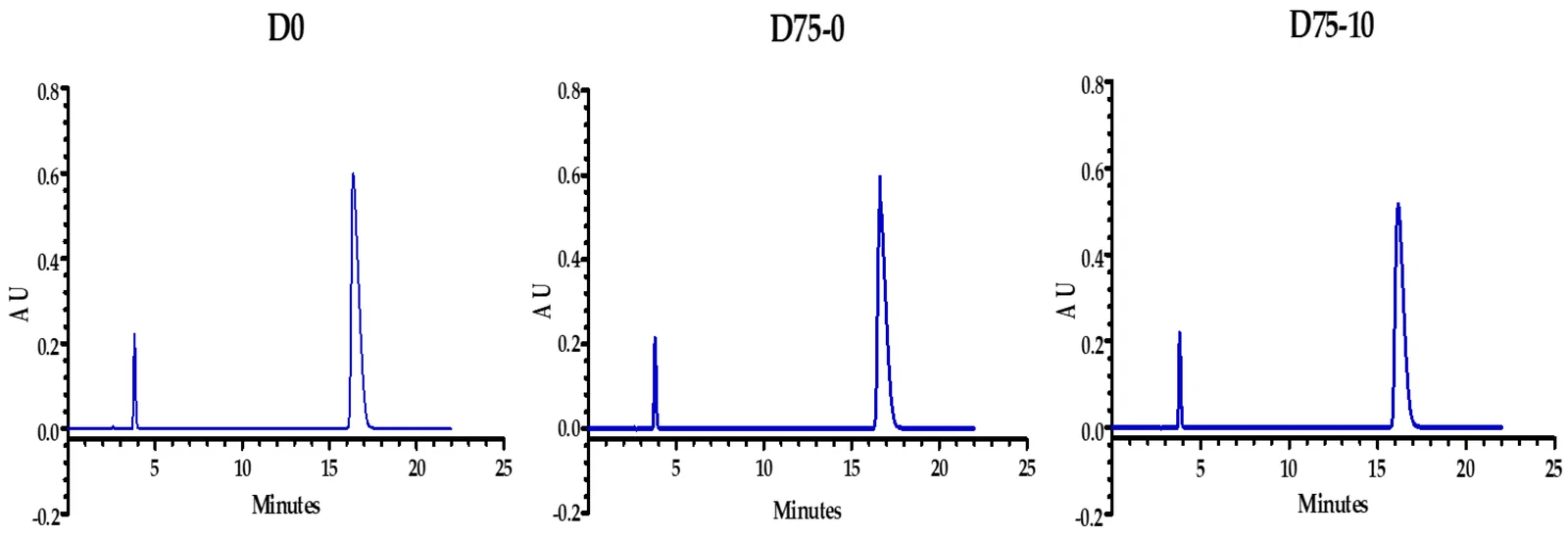
Chromatograms of oseltamivir 15 mg/mL oral solution in amber glass bottles under refrigerated conditions on days D0, D75-0, and D75-10 (D + Number1 or D + Number1-Number2; Number 1 = day of thawing and transfer to refrigerated group; Number 2 = day of test in each refrigerated group).

**Figure 7:**
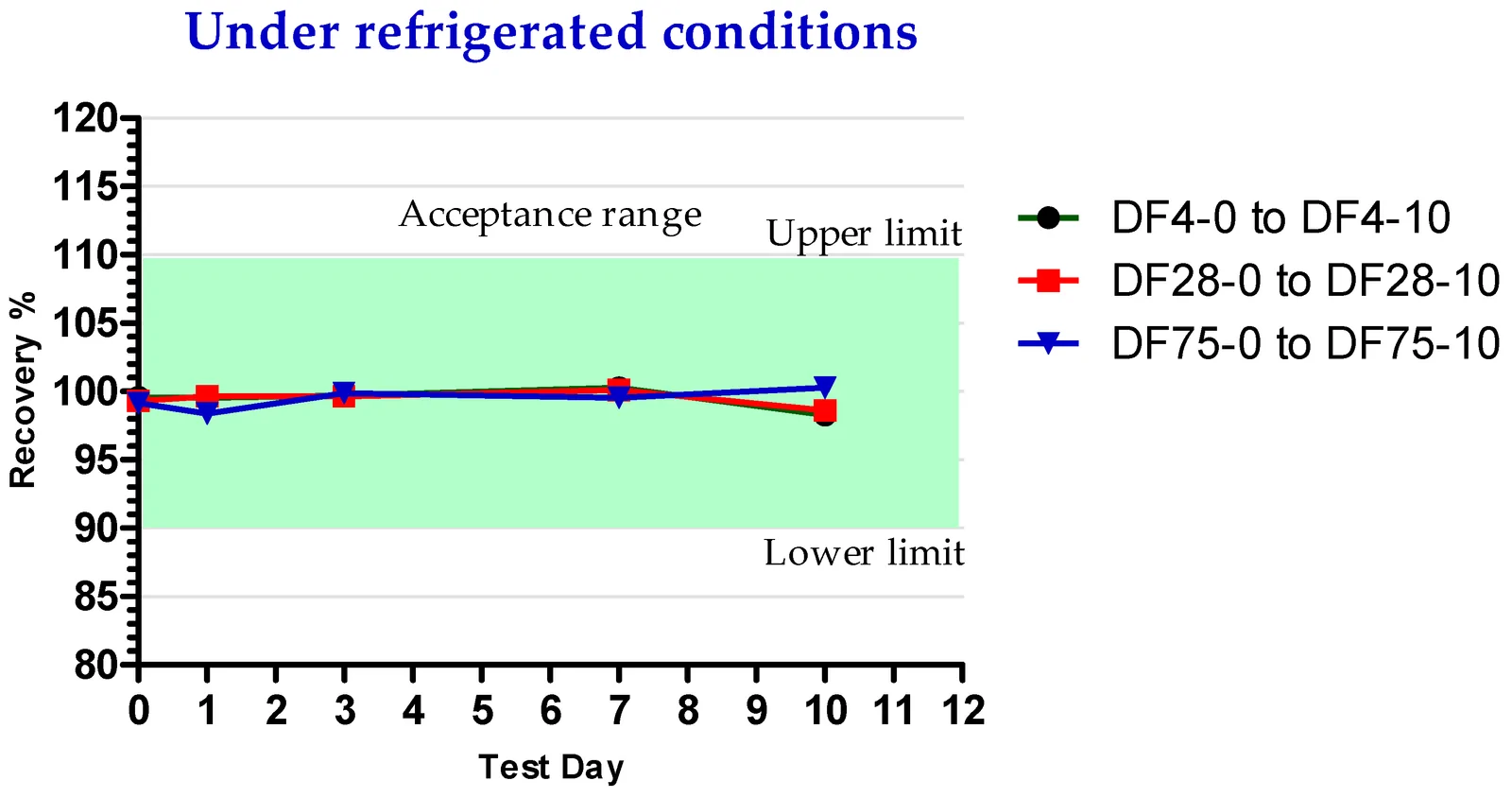
Evolution of percentage recovery of oseltamivir 15 mg/mL oral solution in amber glass bottles during the 10 days of refrigeration (DF4-0, DF28-0, and DF75-0 = test days 4, 28, and 75 after thawing and before moving to the refrigerated group).

The stability data in Table 5 correspond to oseltamivir oral solution samples stored under freezing conditions for 0, 4, 28, 42, and 75 days, followed by storage at 2–8 C for 10 days after thawing. Detailed stability data are provided in Table S6 of the Supplementary Materials. Representative chromatograms obtained at D0 and on the test days D75-0 and D75 for the formulation stored under refrigeration conditions are presented in Figure 6. The concentration profiles of oseltamivir oral solution under three freezing storage periods and during subsequent refrigerated storage for up to 10 days after thawing are shown in Figure 7.

No significant changes were monitored in pH, color, visible particulates, turbidity, and crystallization throughout the study.

The 15 mg/mL oseltamivir oral solution in amber glass bottles was consequently considered physically and chemically stable for up to 10 days under refrigerated storage after thawing.

#### 3.2.3. Evaluation of Microbiological Stability

On day 10, aerobic, anaerobic, and fungal cultures of the oseltamivir oral solution in amber glass bottles under refrigeration yielded negative results for all analyzed samples.

## 4. Discussion

The proactive preparation and long-term storage of oseltamivir phosphate liquid formulations represent a strategic pillar in contingency plans for influenza pandemics. While the DAC/NRF monograph for oseltamivir 15 mg/mL oral solution establishes the compounding guidelines, previously reported stability limits are restricted to refrigerated or room temperature conditions. The study by Albert and Bockshom evaluated the standardized preparation from the DAC/NRF at room temperature (25 °C) and under refrigeration (6 °C) for 84 days. The study considered the assay of oseltamivir content and the appearance of degradation products such as oseltamivir isomer I, whose content in liquid oseltamivir preparations is limited to 0.5% for toxicological reasons, as well as isomer II and oseltamivir carboxylate. It concluded that the preparation was stable for up to 84 days under refrigeration and for up to 46 days at room temperature when purified water was used [13]. In the remaining studies reviewed, the oral preparation was mainly made from capsules and commercial tablets (Tamiflu^TM^), using cherry syrup, Ora-Sweet^TM^ SF, SyrSpend SF^TM^, or SyrSpend SF PH^TM^ as the vehicle. The stability of these oseltamivir oral suspensions at 15 mg/mL ranged from 5 to 13 days at room temperature and from 30 to 90 days under refrigeration, both under light protection [18,27,28,29].

It is also noteworthy that the commercially available oseltamivir phosphate oral suspensions (NDC codes: 72303-0851-1, 64380-879-75, 31722-108-60, and 68788-8123-6) are labeled “Do not freeze” [30,31,32,33]. This instruction is consistent with the storage conditions approved by the U.S. Food and Drug Administration for constituted oseltamivir oral suspensions, for which storage is limited to refrigeration or controlled room temperature for defined periods. The FDA-approved product is a 6 mg/mL suspension reconstituted from a powder formulation and therefore differs from the 15 mg/mL aqueous solution investigated in the present study in terms of concentration, excipient composition, dosage form, and packaging configuration. The “Do not freeze” statement should therefore be interpreted as a product-specific regulatory storage restriction rather than as evidence that freezing is intrinsically unsuitable for all aqueous oseltamivir formulations. In the absence of supporting freeze–thaw stability data, freezing may pose potential risks, including freeze-concentration of the dissolved components, local pH changes, precipitation or irreversible sedimentation, loss of dose homogeneity after thawing, and mechanical stress on the container. These considerations provide a plausible rationale for excluding freezing from the labeled storage conditions of the commercial product. However, the FDA labeling does not establish that any one of these mechanisms is solely responsible for the restriction; rather, the instruction reflects the storage conditions supported by the product-specific stability package.

The present study evaluates for the first time an alternative conservation scheme combining a prolonged freezing phase at −20 °C (75 days) followed by an in-use phase under refrigeration at 5 °C (10 days). To contextualize the physical and chemical viability observed in this formulation, it is important to know that the freezing of this aqueous solution could induce the separation of pure water into ice crystals, actively segregating the API and the preservative (sodium benzoate) into a non-frozen residual liquid phase (the freeze-concentration phenomenon) [34,35]. This dramatic increase in local solute concentration could trigger extreme local pH fluctuations [36,37], accelerate the kinetics of hydrolytic degradation or acyl migration, and promote crystal nucleation or irreversible sedimentation (caking) [38] upon thawing, potentially compromising the effective dose and the mechanical integrity of the container [39]. Despite the risks implicit in this preparation, the experimental results demonstrated that the formulation exhibits outstanding physical, chemical, and microbiological stability throughout the evaluated 85-day period. The molecular and physical mechanisms that could explain the formulation’s resilience against these stress factors could be the following:

• The extreme temperature reduction to −20 °C drastically decreases the thermal energy and molecular mobility of the system. According to the Arrhenius equation, this temperature drop reduces the reaction rate constant to negligible levels and could effectively block the kinetics of both hydrolysis and isomerization. This is consistent with the chemical recovery of the drug, which remained virtually unchanged within the strict acceptance limits of 90–110% during both the 75-day frozen storage (D75) and the subsequent 10-day refrigeration period (D75-10).
• The stability of local pH: A shift in the microenvironmental pH of the residual liquid phase due to the fractional precipitation of solutes during freezing often accelerates the degradation of pH-sensitive drugs. For oseltamivir phosphate, the optimum stability profile is situated at a narrow pH of approximately 4.0. The experimental results revealed that the pH range of the solution remained remarkably stable during frozen storage and subsequent refrigeration. This range, being close to the drug’s maximum stability point, prevented the significant acid- or base-catalyzed degradation that typically occurs when the pH shifts toward extreme values.
• Homogeneity, Redispersibility, and Crystallization: Ice crystal nucleation and growth can force suspended particles together, potentially inducing irreversible compact sedimentation (caking). Additionally, there is a risk that thawing might disrupt the physical homogeneity of the dose. However, oseltamivir phosphate is a highly water-soluble molecule. Gentle manual rocking of the solution for two minutes after thawing proved sufficient to achieve complete and homogeneous redispersion of the system, with all replicates classified as colorless, transparent, and without visible particulates. Moreover, phase-contrast microscopic evaluations further confirmed the complete absence of crystals or particulates larger than 10 µm/mL.
• Container Mechanical Integrity and Microbiological Stability: The phase transition of water to ice involves a volume expansion of approximately 9%, exerting severe hydrostatic and mechanical stress on the container walls. If the glass container is not properly designed or lacks sufficient headspace for expansion, breakage or loss of closure integrity is inevitable. In this protocol, packaging a net volume of 50 mL into the 125 mL Type II amber glass bottles provided adequate headspace (representing over 50% of the total bottle capacity) to absorb the thermal expansion. This successfully prevented any mechanical failure or microcracks that could compromise the container closure integrity. Consequently, microbiological cultures for aerobic, anaerobic, and fungal microorganisms performed after four cycles of refrigerated storage, equilibration to room temperature, and bottle opening for sample withdrawal on the final day of the study (Day 85) yielded negative results for all analyzed batches. These results confirm that sodium benzoate retains its antimicrobial preservative activity intact after frozen storage, aligning with its documented stability under extreme environmental conditions in the pharmaceutical literature [13].

It must be acknowledged that a limitation of this study lies in the fact that the validated isocratic chromatographic method focused strictly on the quantification of the active ingredient recovery (oseltamivir phosphate), without performing direct, individual tracking of minor degradation products such as oseltamivir carboxylate, isomer I, or isomer II. In liquid oseltamivir preparations, the toxicological limit for the N,N-acyl-migrated degradant (isomer I) is strictly capped at 0.5%. Although the solution’s pH remained well-controlled near the drug’s maximum stability point—minimizing the theoretical rate of degradation—future long-term stability studies under frozen conditions (e.g., 6 to 12 months) should incorporate the simultaneous HPLC quantification of these specific impurities and formal preservative efficacy testing to fully validate the protocol for pandemic stockpiling in hospital pharmacies.

## 5. Conclusions

Under the specific conditions evaluated, the oseltamivir phosphate 15 mg/mL oral solution prepared from API and preserved with sodium benzoate remained within the predefined chemical acceptance limits during 75 days of frozen storage and for an additional 10 days of refrigerated storage after thawing. No relevant physical changes were observed, and microbiological testing performed at the end of the study was negative. These findings support the feasibility of this storage strategy for hospital pharmacy preparation.

## Figures and Tables

**Table 1:** Composition of oseltamivir 15 mg/mL oral solution per 100 mL.

Ingredients	Quantity
Oseltamivir phosphate *	1.971 g
Sodium benzoate	0.1 g
Purified water	to 100 mL

* Equivalent to 1.5 g of oseltamivir.

**Table 4:** Physicochemical results of oseltamivir 15 mg/mL oral solution in amber glass bottles under freezing conditions.

Parameter	D0	D4	D28	D75
R %	100	99.583 ± 0.001	99.332 ± 0.007	99.165 ± 0.003
pH	4.938 ± 0.037	5.011 ± 0.002	5.032 ± 0.003	5.047 ± 0.005
Color	Colorless	Colorless	Colorless	Colorless
Appearance	Transparent	Transparent	Transparent	Transparent
Visible Particulates	Absence	Absence	Absence	Absence
Crystals ≥ 10 µm/mL	0	0	0	0

Results are expressed as mean ± SD (standard deviation) of triplicate determinations; AVR% = average recovery percentage; D0 = Day 0 of analysis; D4 = Day 4 of analysis; D28 = Day 28 of analysis; D75 = Day 75 of analysis.

**Table 5:** Physicochemical results of oseltamivir 15 mg/mL oral solution in amber glass bottles under refrigeration conditions, after thawing.

DF	Test Day	Total Days of Study	SC	AVR%	pH	Color	Appearance	Visible Particulates	Crystals ≥ 10 µm/mL
	**D0**	0		100	4.938 ± 0.0 37	Colorless	Transparent	Absence	0
4	D4-0	4	F	99.583 ± 0.001	5.011 ± 0.002	Colorless	Transparent	Absence	0
	D4-1	5	R	99.555 ± 0.005	5.014 ± 0.007	Colorless	Transparent	Absence	0
	D4-3	8	R	99.722 ± 0.002	5.020 ± 0.003	Colorless	Transparent	Absence	0
	D4-7	11	R	100.278 ± 0.012	5.007 ± 0.008	Colorless	Transparent	Absence	0
	D4-10	14	R	98.275 ± 0.005	5.023 ± 0.002	Colorless	Transparent	Absence	0
28	D28-0	28	F	99.332 ± 0.007	5.032 ± 0.003	Colorless	Transparent	Absence	0
	D28-1	29	R	99.666 ± 0.002	5.007 ± 0.008	Colorless	Transparent	Absence	0
	D28-3	31	R	99.665 ± 0.006	5.033 ± 0.002	Colorless	Transparent	Absence	0
	D28-7	35	R	100.111 ± 0.003	5.030 ± 0.003	Colorless	Transparent	Absence	0
	D28-10	38	R	98.609 ± 0.002	5.032 ± 0.004	Colorless	Transparent	Absence	0
75	D75-0	75	F	99.165 ± 0.003	5.047 ± 0.005	Colorless	Transparent	Absence	0
	D75-1	76	R	98.386 ± 0.002	5.035 ± 0.005	Colorless	Transparent	Absence	0
	D75-3	78	R	99.889 ± 0.003	5.027 ± 0.006	Colorless	Transparent	Absence	0
	D75-7	82	R	99.555 ± 0.004	5.023 ± 0.004	Colorless	Transparent	Absence	0
	D75-10	85	R	100.278 ± 0.003	5.033 ± 0.003	Colorless	Transparent	Absence	0

Results are expressed as mean ± SD (standard deviation) of triplicate determinations; AVR% = average recovery percentage; D0 = Day 0 of the test; DF = days of freezing before thawing; SC = storage condition (F = frozen; R = refrigerated); Test day: day of sampling (D + Number1-Number2; Number 1 = day of thawing and transfer to refrigerated group; Number 2 = day of test in each refrigerated group).

## Data Availability

The data presented in this study are available upon request from the corresponding author.

## Supplementary Materials

The following supporting information can be downloaded at https://www.mdpi.com/article/10.3390/pharmaceutics18091130/s1, Table S1: Peak area and oseltamivir concentration for making the calibration curve; Table S2: Data from intra-day repeatability of oseltamivir samples at 67%, 100%, and 133% of the target concentration of 0.6 mg/mL; Table S3: Data from inter-day repeatability of oseltamivir samples at 67%, 100%, and 133% of the target concentration of 0.6 mg/mL; Table S4: Data from accuracy of oseltamivir samples at 67%, 100%, and 133% of the target concentration of 0.6 µg/mL; Table S5: Average Recovery% of three determinations on the day of the test and pH of oseltamivir 15 mg/mL oral solution in amber glass bottles under freezing conditions; Table S6: Average Recovery% of three determinations on the day of the test and pH of oseltamivir 15 mg/mL oral solution in amber glass bottles under refrigerated conditions; Figure S1: Linearity between the peak area and oseltamivir concentration for making the calibration curve.

## Abbreviations

The following abbreviations are used in this manuscript:

API	Active pharmaceutical ingredient
FSG	Frozen stability group
G-	Gram-negative bacteria
G+	Gram-positive bacteria
DAC/NRF	German National Formulary
GPPMHPS	Guide to good preparation practices for medicines in hospital pharmacy services
ICH	International Council for Harmonisation of Technical Requirements for Pharmaceuticals for Human Use
LOD	Limit of detection
LOQ	Limit of quantification
PP	Polypropylene
RSD%	Relative standard deviation
RSG	Refrigerated stability group
SC	Storage condition
