# Supplementary material for: Stability Study of a Frozen Oseltamivir 15 mg/mL Oral Solution in Amber Glass Bottles
Source: Pharmaceutics. 2026 Sep 8;18(9):1130. doi: 10.3390/pharmaceutics18091130 (PMC13610113; doi:10.3390/pharmaceutics18091130)
Supplement: Supplementary file 1 [file pharmaceutics-18-01130-s001.zip › pharmaceutics-4482547-supplementary.pdf]

Supplementary Materials

Supplementary Table S1. Peak area and oseltamivir point concentrations for creating the calibration curve.

Point	Concentration (mg/mL)	Peak area (μV*sec)
1	0.3	7.187E+06
1	0.3	7.124E+06
1	0.3	7.203E+06
1	0.3	7.126E+06
1	0.3	7.049E+06
2	0.4	9.470E+06
2	0.4	9.507E+06
2	0.4	9.516E+06
2	0.4	9.503E+06
2	0.4	9.583E+06
3	0.5	1.180E+07
3	0.5	1.216E+07
3	0.5	1.177E+07
3	0.5	1.196E+07
3	0.5	1.180E+07
4	0.6	1.441E+07
4	0.6	1.423E+07
4	0.6	1.444E+07
4	0.6	1.415E+07
4	0.6	1.412E+07
5	0.7	1.678E+07
5	0.7	1.6747E+07
5	0.7	1.651E+07
5	0.7	1.671E+07
5	0.7	1.697E+07
6	0.8	1.906E+07
6	0.8	1.895E+07
6	0.8	1.888E+07
6	0.8	1.924E+07
6	0.8	1.917E+07
7	0.9	2.170E+07
7	0.9	2.135E+07
7	0.9	2.176E+07
7	0.9	2.132E+07
7	0.9	2.142E+07
8	1	2.348E+07
8	1	2.383E+07
8	1	2.353E+07
8	1	2.367E+07
8	1	2.345E+07

Supplementary Figure S1. Linearity between the peak area and 8 concentration values (Table ST1) of oseltamivir for creating the calibration curve.

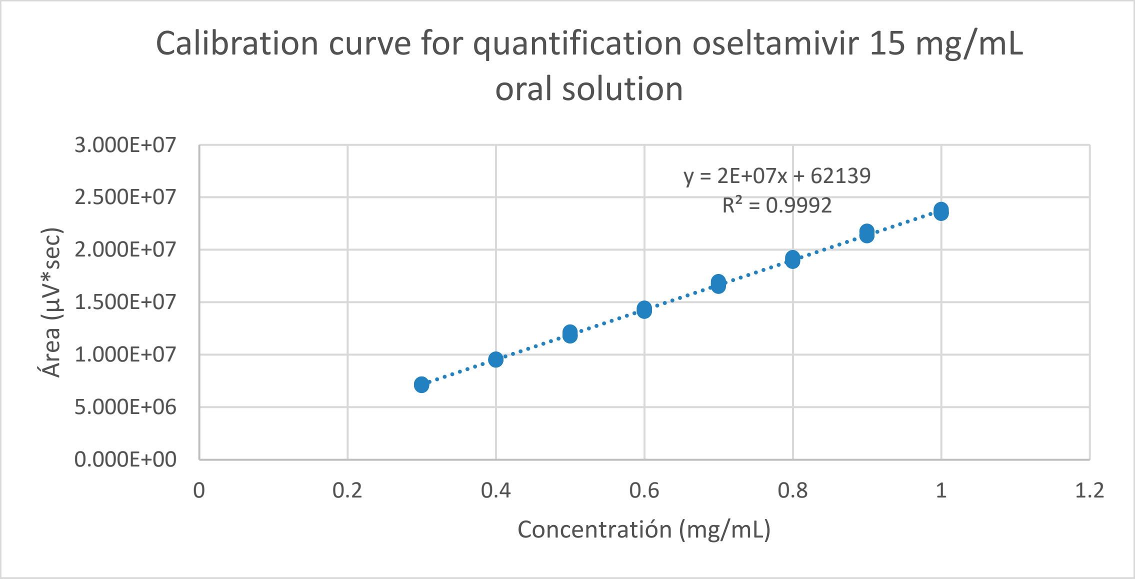

Supplementary Table S2. Data from intra-day repeatability of the quantification method using oseltamivir 15 mg/mL oral solution.

Target concentration (mg/mL)	Estimated concentration (µg/mL)	Mean	SD	RSD%	RSD% mean	Accuracy%	Accuracy% mean
0.4 (67%)	0.398<br>0.399<br>0.398<br>0.400<br>0.400	0.399	0.001	0.264	0.461	99.425<br>99.723<br>99.574<br>100.025<br>100.003	99.750
0.6 (100%)	0.602<br>0.596<br>0.601<br>0.595<br>0.599	0.599	0.003	0.522		100.381<br>99.370<br>100.152<br>99.147<br>99.916	99.793
0.8 (133%)	0.802<br>0.799<br>0.797<br>0.808<br>0.796	0.800	0.005	0.596		100.199<br>99.894<br>99.642<br>100.983<br>99.466	100.038

RSD%: relative standard deviation; SD: standard deviation.

Supplementary Table S3. Data from inter-day repeatability of the quantification method using oseltamivir 15 mg/mL oral solution.

Target concentration (mg/mL)	Estimated concentration (μg/mL)	Day	Mean	SD	RSD (%)	RSD% mean	Accuracy %	Accuracy% mean
0.4 (67%)	0.398	1	0.399	0.002	0.378	0.566	99.462	99.848
	0.401	1					100.187	
	0.398	1					99.613	
	0.402	2					100.386	
	0.398	2					99.526	
	0.400	2					99.892	
	0.397	3					99.363	
	0.401	3					100.301	
	0.399	3					99.728	
	0.400	4					100.074	
	0.398	4					99.406	
	0.401	4					100.234	
0.6 (100%)	0.596	1	0.600	0.004	0.712		99.292	100.020
	0.605	1					100.857	
	0.599	1					99.782	
	0.602	2					100.250	
	0.595	2					99.168	
	0.606	2					101.016	
	0.600	3					99.935	
	0.596	3					99.383	
	0.603	3					100.541	
	0.601	4					100.100	
	0.594	4					99.005	
	0.605	4					100.910	
0.8 (133%)	0.796	1	0.802	0.005	0.608		99.506	100.232
	0.807	1					100.889	
	0.798	1					99.748	
	0.801	2					100.162	
	0.809	2					101.072	
	0.795	2					99.390	
	0.804	3					100.516	
	0.799	3					99.859	
	0.806	3					100.747	
	0.797	4					99.594	
	0.803	4					100.318	
	0.808	4					100.986	

RSD%: relative standard deviation; SD: standard deviation.

Supplementary Table S4. Accuracy data for the quantification method of oseltamivir 15 mg/mL oral solution at 67%, 100%, and 133% of the target concentration of 0.6 µg/mL (recovery expressed as a percentage of recovery of the theoretical value (100%)).

Target concentration (mg/mL)	Estimated concentration (mg/mL)	Recovery%	Mean	SD	RSD%
0.4 (67%)	0.398	99.600	100.045	0.496	0.496
	0.401	100.300			
	0.399	99.775			
	0.401	100.125			
	0.398	99.425			
	0.402	100.450			
0.6 (100%)	0.599	99.783			
	0.603	100.517			
	0.596	99.267			
	0.604	100.700			
	0.597	99.533			
	0.602	100.250			
0.8 (133%)	0.800	99.975			
	0.805	100.650			
	0.797	99.588			
	0.808	101.013			
	0.801	100.175			
	0.798	99.688			

Recovery%: percentage of recovery of the theoretical value (100%); RSD%: relative standard deviation; SD: standard deviation.

Supplementary Table S5. Average Recovery% of three determinations on the day of the test and pH of frozen oseltamivir 15 mg/mL oral solution.

Test Day	C1 (mg/mL)	C2 (mg/mL)	C3 (mg/mL)	AVC	R%	pH
D0	0.595	0.597	0.605	0.599	100.000	4.909
D4	0.599	0.598	0.598	0.598	99.889	5.011
D11	0.589	0.590	0.591	0.590	98.497	5.017
D18	0.588	0.591	0.593	0.591	98.609	5.007
D28	0.590	0.598	0.595	0.594	99.221	5.032
D42	0.588	0.589	0.590	0.589	98.331	5.003
D60	0.593	0.591	0.593	0.592	98.887	5.026
D75	0.595	0.592	0.595	0.594	99.165	5.047

AVC: average value concentrations of C1, C2 and C3; D: day of the test; R%: recovery percentage value of AVC in each test day with respect to AVC test day D0.

Supplementary Table S6. Average Recovery% of three determinations on the day of the test and pH of oseltamivir 15 mg/mL oral solution under refrigerated conditions after thawing.

DF	Test Day	Total days of study	C1 (mg/mL)	C2 (mg/mL)	C3 (mg/mL)	AVC	R%	pH
	D0	0	0.595	0.597	0.605	0,599	100.000	4.938
4	D4-0	4	0.599	0.598	0.598	0.598	99.833	5.011
	D4-1	5	0.593	0.597	0.599	0.596	99.499	5.015
	D4-3	8	0.597	0.599	0.596	0.597	99.666	5.021
	D4-7	11	0.596	0.609	0.597	0.601	100.334	5.007
	D4-10	14	0.592	0.588	0.586	0.589	98.331	5.023
11	D11-0	11	0.589	0.590	0.591	0.590	98.497	5.017
	D11-1	12	0.599	0.593	0.595	0.596	99.499	4.989
	D11-3	14	0.598	0.599	0.602	0.600	100.167	5.016
	D11-7	18	0.596	0.596	0.604	0.599	100.000	4.997
	D11-10	21	0.595	0.589	0.591	0.592	98.831	5.023
18	D18-0	18	0.588	0.591	0.593	0.591	98.664	5.007
	D18-1	19	0.596	0.594	0.595	0.595	99.332	5.033
	D18-3	21	0.596	0.595	0.605	0.599	100.000	5.030
	D18-7	25	0.597	0.595	0.592	0.595	99.332	5.034
	D18-10	35	0.586	0.589	0.589	0.588	98.164	5.026
28	D28-0	28	0.590	0.598	0.595	0.594	99.165	5.032
	D28-1	29	0.597	0.596	0.598	0.597	99.666	5.030
	D28-3	32	0.594	0.596	0.601	0.597	99.666	5.033
	D28-7	35	0.598	0.602	0.599	0.600	100.167	5.054
	D28-10	38	0.592	0.591	0.589	0.591	98.664	5.022
42	D42-0	42	0.588	0.589	0.590	0.589	98.331	5.003
	D42-1	43	0.588	0.589	0.603	0.593	98.998	5.017
	D42-3	45	0.597	0.599	0.601	0.599	100.000	5.026
	D42-7	49	0.599	0.598	0.596	0.598	99.833	5.023
	D42-10	52	0.599	0.601	0.598	0.599	100.000	5.040
60	D60-0	60	0.593	0.591	0.593	0.592	98.831	5.026
	D60-1	61	0.595	0.597	0.605	0.593	98.998	5.026
	D60-3	63	0.593	0.589	0.596	0.601	100.334	5.031
	D60-7	67	0.599	0.602	0.601	0.596	99.499	5.039
	D60-10	70	0.593	0.599	0.595	0.600	100.167	5.027
75	D75-0	75	0.595	0.592	0.595	0.594	99.165	5.047
	D75-1	76	0.588	0.590	0.590	0.589	98.331	5.036
	D75-3	78	0.600	0.598	0.597	0.598	99.833	5.027
	D75-7	82	0.595	0.595	0.599	0.596	99.499	5.023
	D75-10	85	0.599	0.601	0.602	0.601	100.334	5.033

AVC: average value concentrations of C1, C2, and C3; DF: days of freezing before thawing; R%: recovery percentage value of AVC in each test day with respect to the AVC test day D0; Test day: day of sampling (D+Number1-Number2; Number 1 = day of thawing and transfer to refrigerated group; Number 2 = day of test in each refrigerated group).
